# LncRNA and its role in gastric cancer immunotherapy

**DOI:** 10.3389/fcell.2023.1052942

**Published:** 2023-02-16

**Authors:** Qiang Zhang, Chuanchi Wang, Yan Yang, Ruihan Xu, Ziyun Li

**Affiliations:** ^1^ Department of Digestive endoscopy, Jiangsu Province Hospital of Traditional Chinese Medicine, Affiliated Hospital of Nanjing University of Chinese Medicine, Nanjing, Jiangsu, China; ^2^ Xin-Huangpu Joint Innovation Institute of Chinese Medicine, Guangzhou, Guangdong, China; ^3^ China Science and Technology Development Center of Chinese Medicine, Beijing, China; ^4^ The Comprehensive Cancer Centre of Nanjing Drum Tower Hospital, The Affiliated Hospital of Nanjing University Medical School, Nanjing, Jiangsu, China; ^5^ Acupuncture and Tuina college, Nanjing University of Chinese Medicine, Nanjing, Jiangsu, China

**Keywords:** gastric cancer, lncRNA, immunity, therapy, mechanism

## Abstract

Gastric cancer (GC) is a potential dominant disease in tumor immunotherapy checkpoint inhibitors, and adoptive cell therapy have brought great hope to GC patients. However, only some patients with GC can benefit from immunotherapy, and some patients develop drug resistance. More and more studies have shown that long non-coding RNAs (lncRNAs) may be important in GC immunotherapy’s prognosis and drug resistance. Here, we summarize the differential expression of lncRNAs in GC and their impact on the curative effect of GC immunotherapy, discuss potential mechanisms of activity in GC immunotherapy resistance regulated by lncRNAs. This paper reviews the differential expression of lncRNA in GC and its effect on immunotherapy efficacy in GC. In terms of genomic stability, inhibitory immune checkpoint molecular expression, the cross-talk between lncRNA and immune-related characteristics of GC was summarized, including tumor mutation burden (TMB), microsatellite instability (MSI), and Programmed death 1 (PD-1). At the same time, this paper reviewed the mechanism of tumor-induced antigen presentation and upregulation of immunosuppressive factors, as well as the association between Fas system and lncRNA, immune microenvironment (TIME) and lncRNA, and summarized the functional role of lncRNA in tumor immune evasion and immunotherapy resistance.

## 1 Introduction

Gastric cancer (GC) has become the fifth commonest cancer in 2020, with a 5.6% incidence rate and a 7.7% death rate, according to the updated research (Sung et al., 2021). As a kind of cancer with poor prognosis, there are several risk factors, such as *helicobacter pylori* infection, age and high salt intake (Smyth et al., 2020). Apart from common surgeries, including surgical or endoscopic resection, adjuvant and perioperative chemotherapy, immunotherapy, especially immunotherapy checkpoint inhibitors (ICI) therapy, is now established as an essential strategy for chemo refractory GC therapy (Kang et al., 2017; Wang et al., 2021a). Besides, the use of cytotoxic immunocytes and gene transferred vaccines also grow rapidly (Joshi and Badgwell, 2021). Unfortunately, GC has variable responsiveness to immunotherapy due to the heterogeneity of the disease, which is a great challenge to this cancer treatment.

Many factors influence the efficacy of immunotherapy for that gastric tumorigenesis involves a series of genetic, epigenetic, and epitranscriptomic alterations (Xie et al., 2021). Currently, long non-coding RNAs (lncRNAs) has more than 200 nucleotides at length and takes emerging roles in the immunosuppressive tumor microenvironment (Xiao et al., 2022a). As a cluster of RNAs regulating multiple protein-coding genes, the alterations in lncRNAs expression and their mutations promote tumorigenesis and metastasis (Bhan et al., 2017; Luo et al., 2020). When it comes to GC, the abnormal expressions of lncRNAs are strongly related to its chemoresistance, drug resistance, and immunotherapy (Wei et al., 2020a; Yuan et al., 2020). However, definite mechanisms under the interplay between lncRNA and GC are still unclear.

In the following paragraphs, we summarize the interferences between immunological characteristics of GC and lncRNAs. Meanwhile, we conclude the factors associated with the response to immunotherapy of GC, focusing on the functional roles of lncRNAs in tumor immune evasion and immunotherapy resistance emphatically.

## 2 LncRNAs have superior values in optimizing patients’ selection for ICIs therapy and predicting patients’ outcomes of ICI therapy

**FIGURE 1:**
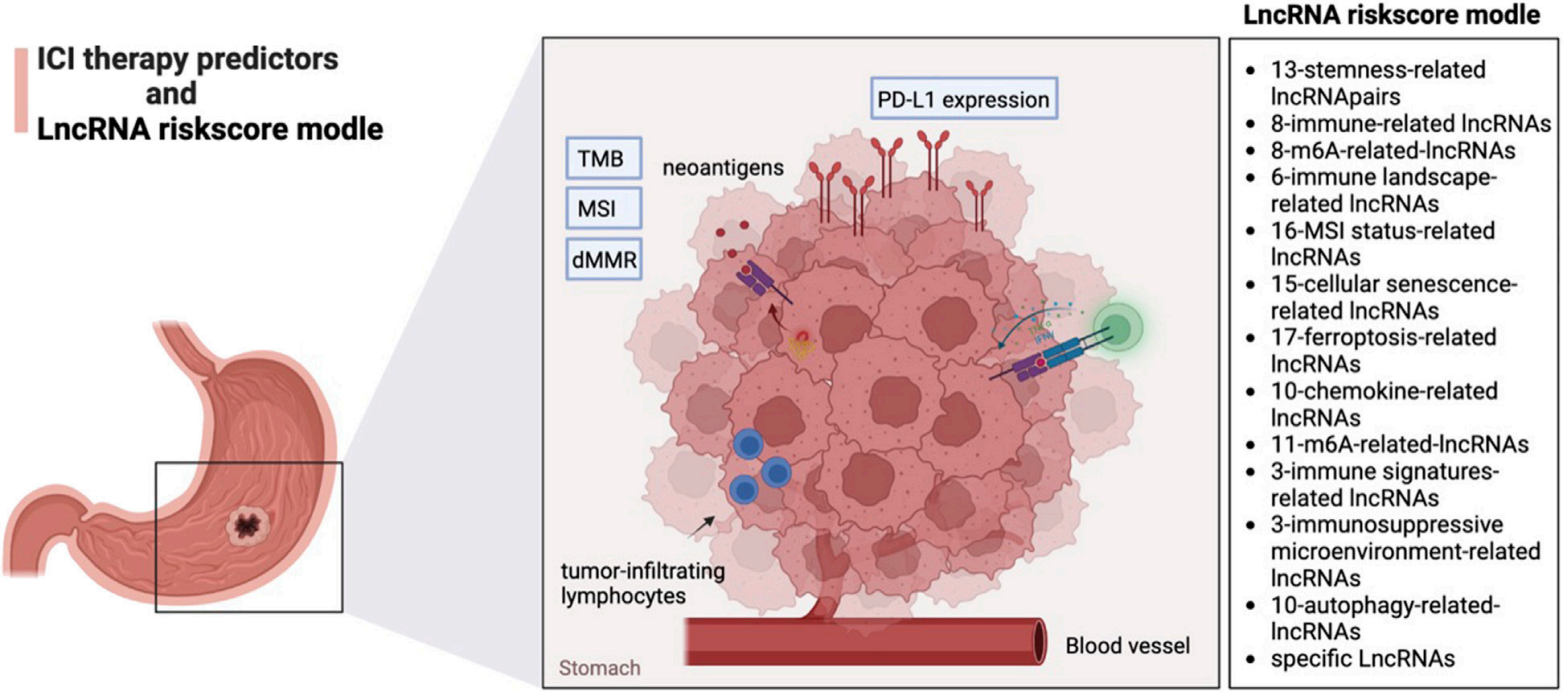
The lncRNAs risk models in guiding the treatment of ICI.

Programmed death 1 (PD-1) ligand (PD-L1) (CD274) expression, tumor mutation burden (TMB), and microsatellite instability (MSI) status of tumor tissue are potential predictors of anti-PD-1 treatment response (Schreiber et al., 2011; Galon and Bruni, 2019). The expression of PD-L1 was correlated with the tumor infiltration level, TMB, MSI, and dMMR of different types of cancers (Dai et al., 2021). Many researchers have investigated the relationship between various biomarkers, including TMB, MSI, PD-L1 expression, *etc.*, and the lncRNAs risk models in guiding the treatment of ICI (Figure 1).

### 2.1 Genome stability and lncRNAs risk models

As a widely used indicator of tumor immunogenicity, TMB is a quantifiable biomarker affecting immune checkpoint inhibitors. It reflects the number of mutations in a tumor cell, usually expressed as mutations per megabase. In other words, TMB is a statistic and calculation of the number of tumor mutations (Alexandrov et al., 2013). The higher the value of TMB, the more mutations that can produce neoantigens, the higher the immune response rate, and the better the effect of tumor immunotherapy. At present, TMB detection methods are mainly based on high-throughput sequencing platforms of whole exon sequencing and targeted Panel sequencing (Pardoll, 2012). It is worth noting that the definitions of TMB-H and TMB-L are not set in stone (Carbone et al., 2017; Goodman et al., 2017; Marabelle et al., 2020). In the current studies, TMB-H (≥10 mutations/MB) and TMB-L (<10 mutations/MB) are mainly used to distinguish them (Marabelle et al., 2020). For the distribution of tumor patients with low, medium, and high load TMB expression, some studies believe that the proportion of low TMB tumor patients is about 50%, the proportion of medium TMB tumor patients is about 40%, and the proportion of high TMB tumor patients is only about 10% (Goodman et al., 2017).

In a targeted sequencing study of 529 Chinese patients with gastric adenocarcinoma, the genetic mutations of TMB-H GC patients were mainly in ARID1A, KMT2D, RNF43, TGFBR2 and CIC. The gene mutations in TMB-L GC patients were mainly in ERBB2, CCNE1, CDK12 and CCND1 (Yu et al., 2021). There have also been other reports that mutations in the LRP1B gene are so prominent, in Chinese GC patients with high TMB (Zhang et al., 2021).

Many researchers have built the lncRNAs risk score models in TMB in guiding the treatment of ICI. A negative correlation is prevalent between cancer stemness and anticancer immunity (Miranda et al., 2019). Jiang Q et al. established a 13-DEsrlncRNA pair-based signature. This study could provide a stemness-related lncRNA signature for survival prediction in GC patients and establish a model with predictive potentials for GC patients’ sensitivity to chemotherapy and immunotherapy. The risk score presented negative correlations with TMB values based on the Spearman correlation analysis. Compared with CTLA4, the results may indicate a better efficacy of this risk score model for PD-1 therapy response prediction (Jiang et al., 2021). Yujiao Wang et al. established a risk model involving 8 immune-related lncRNA (irlncRNA) pairs and found that patients in the high-risk group had lower TMB scores and poorer prognoses (Wang et al., 2021b). Based on lncRNA, Yi Wang et al. selected 8 lncRNAs to build a feature classifier for predicting TMB level, which is associated with the expression of immune checkpoint tumor-infiltrating lymphocytes and microsatellite instability (Wang et al., 2022a).

Microsatellites are simple repeats of 2-6 nucleotides in the DNA genome, also known as Short Tandem Repeat (STR). When the function of MMR is abnormal, the alignment errors in microsatellite replication cannot be corrected. With the accumulation of replication errors, the base composition or sequence length of microsatellite changes, and the genome shows a hypermutant phenotype. It is called MSI. According to the microsatellite state, it can be divided into MSI-H, low microsatellite instability (MSI-L) and microsatellite stability (MSS). The detection method of MSI mainly uses PCR technology to directly amplify the bases of MSI sites, and then uses capillary electrophoresis to analyze the amplified products. This method is currently considered as the gold standard for MSI detection. Five loci “2B3D” (BAT25, BAT26, D2S123, D5S346 and D17S250) were universally detected. The diagnostic criteria were as follows: instability of 2 or more loci was MSI-H; The instability of one locus was microsatellite MSI-L. If all loci are stable, MSS. The updated NCCN guidelines in 2021 will recommend cancer indications for MSI testing, including GC, which explicitly states that MSI sites select 5-site panels (2B3D National Cancer Institute (NCI) Panel and 5-single nucleotide Panel). In addition, the 2021 CSCO guidelines explicitly state the selection of 2B3D as recommended by the NCI for MSI sites.

In GC, lncRNAs associated with MSI mainly include LINC02678, HOXA10-AS, RHOXF1-AS1, AC010789.1, LINC01150, and TGFB2-AS1 (Sun et al., 2021). A model composed of 16 lncRNA features was established to classify MSI status in patients with GC (Chen et al., 2019). Zeng et al. established a risk score model of 15 lncRNAs. The study found a higher proportion of MSI-H in the low-risk group of GC patients (Zeng et al., 2022). Xiao S et al. constructed a 17-ferroptosis-related-lncRNA signature *via* multivariate Cox analysis to divide patients into low- and high-risk groups. The risk score was significantly higher in the MSI-H or MSI subtype, respectively. Meanwhile, TMB was pronounced in the low-risk group and negatively correlated with the risk score (Xiao et al., 2021a). Liang X et al. first constructed a multi-lncRNA risk model composed of 10 chemokine-related lncRNAs based on The Cancer Genome Atlas (TCGA) expression data. The results demonstrated that the lncRNA risk model better predicts patient survival, immune cell infiltration, and immunotherapy effectiveness. The risk score obtained from the risk model is negatively correlated with TMB. Low-risk patients with single positivity for CTLA4 or PD-1 and double positivity for CTLA4+PD-1 had higher immunotherapy scores. The chemokine-related lncRNA risk model could be used to predict the immunotherapy sensitivity of GC (Liang et al., 2021).

Genomic instability-associated lncRNA signature can show a distinct immune landscape and predict prognosis in GC. To further reveal the potential role of lncRNAs in guiding the treatment of ICI. Genomic instability-associated lncRNA risk models were not completely independent. At the molecular level, TMB is associated with deficient mismatch repair (dMMR), high microsatellite instability (MSI-H), and mutations in DNA polymerase correction domains encoding POLE and POLD1 genes (Jardim et al., 2021).

### 2.2 Expression of inhibitory immune checkpoint molecules and lncRNAs risk models

PD-1/PD-L1 is a key member of the immunoglobulin superfamily B7-CD28 co-stimulatory molecules. The PD-1 receptor on the surface of T cells binds to the PD-L1 ligand expressed on the surface of tumor cells, inhibiting the activation and proliferation of T cells, leading to tumor cells produce immune escape (Okazaki and Honjo, 2007). Therefore, PD-1/PD-L1, as a negative immune regulator, is involved in the regulation of various tumor immunity. At the same time, TAMs, tumor-infiltrating lymphocytes, and circulating tumor cells (CTCs) may be related to regulating the expression of PD-L1 and affecting tumor prognosis. TAMs secrete a variety of cytokines, including VEGF, IL-1β, TNF, IL-10, *etc.*, which further attract Tregs, and promote tumor cells to express PD-L1, inhibiting the immune function of T cells. In addition, its associated exosomes interact with tumor cells to further promote tumor cell proliferation, invasion, migration, and angiogenesis (Gordon et al., 2017). Tumor-infiltrating CD4^+^ T cells express high levels of Helios and upregulate PD-1 expression (Toor et al., 2019). In addition, CTCs mediate the expression level of PD-L1 and promote the distant metastasis of tumor cells. It is worth noting that after CTCs constitute circulating colonies, the probability of tumor progression and metastasis increases (Winograd et al., 2020).

ICI therapy has become one of the most popular immunotherapies, which has significantly changed the current pattern of cancer treatment, and PD-1 immunoblockade therapy is one of the most typical representatives. Studies have confirmed that the expression of PD-1 on the surface of tumor cells is significantly increased in tumor tissues, including non-small cell lung cancer, melanoma, kidney cancer, ovarian cancer, colorectal cancer, pancreatic cancer, GC, breast cancer, etc (Brahmer et al., 2012). Therefore, researchers focused on blocking the PD-1/PD-L1 pathway through immunotherapy to inhibit tumor development. Major breakthroughs have occurred in non-small cell lung cancer (Reck et al., 2022) and Hodgkin lymphoma (Reinke et al., 2020). A variety of immune checkpoint inhibitors related to this have been approved by the FDA for immunotherapy of cancer patients.

The expression of PD-L1 has been shown to correlate with response to ICIs in GC (Rizzo et al., 2020). Liangliang Lei et al. identified 11 m6A-related lncRNA pairs associated with GC prognosis. Patients in the low-risk group had more prolonged overall survival *versus* the high-risk group. Infiltration of cancer-associated fibroblasts, endothelial cells, macrophages, particularly M2 macrophages, and monocytes was more severe in high-risk patients than low-risk individuals, who exhibited high CD4^+^ Th1 cell infiltration in GC. Altered expressions of immune-related genes were observed in both groups. PD-1 and LAG3 expressions were higher in low-risk patients than in high-risk patients. Immunotherapy, either single or combined use of PD-1 or CTLA4 inhibitors, had better efficacy in low-risk patients than high-risk patients (Lei et al., 2022). Three lncRNAs (AC022706.1, LINC01871, and AC006033.2) have been identified as associated with GC immunotherapy responses through multi-omics data analysis. At the same time, seven gene mutations (ARID1A, BCOR, MTOR, CREBBP, SPEN, NOTCH4, and TET1) were identified that were associated with the prognosis of GC patients receiving anti-PD-1/PD-L1 immunotherapy (He and Wang, 2020) which suggests that lncRNA may be used for risk stratification in GC patients by anti-PD-1/PD-L1 immunotherapy. In subsequent studies, GC patients were divided into high-risk and low-risk groups based on immune-related lncRNA characteristics. PD-1 and PD-L1 were highly expressed in the high-risk group (Ma et al., 2021a). Other studies have shown similar results, with CD274 (PD-L1), PDCD1 (PD-1), and PDCD1LG2 (PD-L2) significantly upregulated in high-risk groups (Wang et al., 2022b) which helps identify GC patients who might benefit from immune checkpoint therapy.

There were also some studies that screen out specific lncRNAs. ZFPM2-AS1, located in 8q23.1, plays an oncogenic role in several tumors. ZFPM2-AS1 was correlated with several known immune checkpoints, including CTLA4, PD-1, PD-L1, TIGIT, LAG-3, HAVCR2 (TIM-3), and IDO1, in most tumors. ZFPM2-AS1 expression was correlated with TMB and MSI in GAC. NUP107 and C8orf76 were identified as potential target mRNAs of ZFPM2-AS1, ZFPM2-AS, NUP107, and C8orf76 were highly expressed in GC cells (Chen et al., 2022).

## 3 LncRNAs and the mechanism of immunotherapy resistance in GC

### 3.1 Tumor induced antigen presentation

LncRNA is related to the tumor induced antigen presentation. T cells are lymphoid stem cells derived from bone marrow. T cell receptor (TCR) is a specific receptor for T cells to recognize and bind foreign antigens. TCR cannot directly recognize and bind free soluble antigens, but only recognize antigen molecules processed by antigen presenting cells and connected with major histocompatibility complexes (MHC-I and -II) (Gaud et al., 2018). The initiation of tumor immune response begins with the recognition of tumor specific antigens by MHC on the surface of antigen cells (Boyne et al., 2021). Downregulation of antigen presentation mechanism (MHC-I) will inhibit immunogenicity and accelerate immune escape (Di Tomaso et al., 2010). In patients with GC, the expression of MHC-I is generally reduced, and the frequency of downregulation of MHC-I in metastatic cells is higher than that in primary tumor cells (Erdogdu, 2019). Histocompatibility leukocyte antigen complex P5 (HCP5), an important lncRNA located between the MICA and MICB genes in MHC-I region (Zou and Chen, 2021). Targeting lncRNA HCP5 may be a novel approach to enhancing the efficacy of chemotherapy in GC through miR-3619-5p/AMPK/PGC1α/CEBPB axis (Kulski, 2019).

### 3.2 LncRNA is a regulator of immunosuppressive factors in GC

More and more evidence showed that lncRNAs are associated with the upregulation of inhibitory immune checkpoints and thus involved in the development and progression of GC.

LncRNA hypoxia-inducible factor 1 alpha-antisense RNA 2 (HIF1A-AS2) expression is elevated in GC tissues and is associated with poor prognosis of GC (Wen-Ming et al., 2015). It is found that HIF1A-AS2 directly binds to microRNA 429 (miR-429) and negatively regulates miR-429 expression, while MiR-429 directly targets PD-L1and inhibits PD-L1 expression. In summary, HIF1A-AS2 can promote PD-L1 expression by targeting and inhibiting miR-429 (Mu et al., 2021). Urothelial carcinoma-associated 1 (UCA1), also significantly highly expressed in GC tissues, could act as competing endogenous RNA (ceRNA) for miR-193a and miR-214, reducing its transcriptional inhibition of PD-L1, thus promoting PD-L1 expression (Wang et al., 2019). Small nucleolar RNA host gene 15 (SNHG15), a ceRNA of miR-141, increased the expression level of PD-L1 on GC cells, thereby improving the resistance of GC cells to tumor immune response (Dang et al., 2020). Ji Wang et al. demonstrated that targeting the NUT family member 2A antisense RNA 1/miR-376a/Tet-eleven translocation 1/PD-L1 (NUTM2A-AS1/miR-376a/TET1/PD-L1) axis may provide a new strategy for GC diagnosis and treatment (Wang et al., 2020a). Specifically, NUTM2A-AS1 is important in GC drug resistance. MiR-376a targets suppressing the expression levels of downstream TET1 and HIF-1A, while the TET1/HIF-1A complex positively regulated PD-L1. Other lncRNAs, such as prospero homeobox 1-antisense RNA 1 (PROX1-AS1), is also extremely highly expressed in GC and can promote GC cell proliferation and invasion *via* miR-877-5p/PD-L1 axis (Guo et al., 2021).

### 3.3 LncRNAs and fas ligand

Recently, lots of researches proved that lncRNAs and Fas ligand (FasL) are participated in a majority of tumor immune progression. FasL induces programmed cell death through receptors. FasL expression may kill infiltrating lymphocytes and inflammatory cells. On the other hand, some relevant studies have shown that when FasL is expressed in tumors or transplants, the proinflammatory function of FasL may cause rejection (Simon et al., 2002; Newsom-Davis et al., 2009). Some important ceRNAs, such as FAS and hsa-miR-125b-5p, and tumor-infiltrating immune cells might relate to distance metastasis and prognosis of Colon Adenocarcinoma Metastasis (Ai et al., 2020; Chang et al., 2020). In addition, lncRNA cancer susceptibility candidate 7 (CASC7) can downgrade the malignant behaviors of breast cancer with miR-21-5p/FasL axis (Wang et al., 2021c).

### 3.4 LncRNA and immune microenvironment (TIME)

Tumor microenvironment plays an important role in the development of GC. LncRNA is also involved in the regulation of tumor microenvironment (Wang et al., 2022c). Many abnormally expressed lncRNAs have been recognized in GC tissues, which affect the occurrence and prognosis of tumors (Table 1). Moreover, lncRNA regulate immunity in several ways in GC through influencing the polarization of GC-associated macrophages (Xie et al., 2020), the differentiation of natural killer cells (Ou et al., 2021), the regulation of dendritic cells (Demaria et al., 2019) and so on (Xiao et al., 2022b).

**TABLE 1:** LncRNAs involved in TIME of GC disease.

LncRNA	Expression	Pathway	References
LINC00001	Upregulated in GC tissuses	miR-497/MACC1 axis	Ma et al. (2017)
LINC00008	Upregulated in GC tissuses	miR-138/E2F2 axis	Yu et al. (2020)
LINC00023	Downregulated in GC tissuses	p53 signaling pathway	Wei and Wang (2017)
LINC00047	Upregulated in GC tissuses	PI3K/AKT pathway	Zhu et al. (2019)
LINC00152	Upregulated in GC tissuses	EGFR-dependent pathway	Zhou et al. (2015)
LINC00256A	Upregulated in GC tissuses	FAM225A-miR-206-ADAM12 axis	Chen et al. (2021)
LINC00342	Upregulated in GC tissuses	miR-545-5p/CNPY2 axis	Liu and Yang (2021)
LINC00902	Downregulated in GC tissuses	p53 and mIR-23b	Qi et al. (2015)
LINC01082	Upregulated in GC tissuses	suppressed GC cells and PD-L1	Wang et al. (2022f)
LINC01540	Upregulated in GC tissuses	miR-378 to modulate MAPK1 expression	Diao et al. (2018)
LINC-POU3F3	Upregulated in GC tissuses	TGF-beta signal pathway	Xiong et al. (2015)
HIF1A-AS2	Upregulated in GC tissuses	HIF1A-AS2/RP11-366L20.2-miR-29c axis	Nai et al. (2022)

MACC1: metastasis-associated in colon cancer 1; E2F2: E2F transcription factor 2; PI3K: phosphatidylinositol 3-kinase; EGFR: epidermal growth factor receptor; ADAM12: A disintegrin and metalloprotease 12; CNPY2: canopy FGF signaling regulator 2; MAPK1: mitogen-activated protein kinase 1; TGF-beta: transforming growth factor-beta.

Moreover, lncRNA in tumor microenvironment is often used as a prognostic marker of tumors. LncRNA and focal cell apoptosis can be used as a prognostic tool for gastric adenocarcinoma (Wang et al., 2022d). Ferroptosis-related lncRNAs in tumor microenvironment also related to the prognosis of GC (Ma et al., 2021b; Xiao et al., 2021b). LncRNA and immune microenvironment can help us better identify the stage and prognosis of GC (Wang et al., 2020b). LncRNA HOX transcript antisense RNA (lncRNA HOTAIR) promotes the metastasis of GC according to miR-1277-5p and increasing Collagen type V alpha 1 chain (COL5A1) (Wei et al., 2020b). The tumor immune microenvironment and prognosis of N6-methyladenosine (m6A) related lncRNA in GC (Wang et al., 2022e). Meanwhile, ferroptosis-related lncRNA can predict the treatment and prognosis of GC (Li et al., 2022).

## Conclusion

To date, surgeries, cytotoxic immunocytes, gene transferred vaccines and immunotherapy, remain the mainstay of clinical therapies for GC. Especially ICI, has been employed as an essential strategy for refractory GC. LncRNA had superior values in optimizing patients’ selection for ICIs therapy and predicting patients’ outcomes of ICI therapy, as revealed in the following. Firstly, lncRNA risk score models have been built in TMB in guiding the treatment of ICI. Secondly, inhibitory immune checkpoint molecules related lncRNA have been shown to correlate with response to ICIs in GC. The mechanisms of lncRNA in immunotherapy resistance are revealed in the following. On the one hand, lncRNA is related to the tumor induced antigen presentation and a regulator upregulation of immunosuppressive factors in GC. On the other hand, lncRNA can regulate the malignant behaviors *via* FasL axis. In particular, lncRNA regulate immunity in several ways to GC tumor growth and progression. LncRNA in tumor microenvironment is often used as a prognostic marker of tumors. Therefore, lncRNA targeting GC immunotherapy has a wide range of potential applications, the use of lncRNA as a therapeutic target will contribute to the development of novel GC treatment strategies.

## References

[B1] Ai Y. Chen M. Liu J. Ren L. Yan X. Feng Y. (2020). lncRNA TUG1 promotes endometrial fibrosis and inflammation by sponging miR-590-5p to regulate Fasl in intrauterine adhesions. Int. Immunopharmacol. 86, 106703. 10.1016/j.intimp.2020.106703 32599321

[B2] Alexandrov L. B. Nik-Zainal S. Wedge D. C. Aparicio S. A. J. R. Behjati S. Biankin A. V. (2013). Signatures of mutational processes in human cancer. Nature 500, 415–421. 10.1038/nature12477 23945592 PMC3776390

[B3] Bhan A. Soleimani M. Mandal S. S. (2017). Long noncoding RNA and cancer: A new paradigm. Cancer Res. 77, 3965–3981. 10.1158/0008-5472.CAN-16-2634 28701486 PMC8330958

[B4] Boyne C. Lennox D. Beech O. Powis S. J. Kumar P. (2021). What is the role of HLA-I on cancer derived extracellular vesicles? Defining the challenges in characterisation and potential uses of this ligandome. Int. J. Mol. Sci. 22 (24), 13554. 10.3390/ijms222413554 34948350 PMC8703738

[B5] Brahmer J. R. Tykodi S. S. Chow L. Q. Hwu W. J. Topalian S. L. Hwu P. (2012). Safety and activity of anti-PD-L1 antibody in patients with advanced cancer. N. Engl. J. Med. 366, 2455–2465. 10.1056/NEJMoa1200694 22658128 PMC3563263

[B6] Carbone D. P. Reck M. Paz-Ares L. Creelan B. Horn L. Steins M. (2017). First-Line nivolumab in stage IV or recurrent non-small-cell lung cancer. N. Engl. J. Med. 376, 2415–2426. 10.1056/NEJMoa1613493 28636851 PMC6487310

[B7] Chang Z. Huang R. Fu W. Li J. Ji G. Huang J. (2020). The construction and analysis of ceRNA network and patterns of immune infiltration in colon adenocarcinoma metastasis. Front. Cell Dev. Biol. 8, 688. 10.3389/fcell.2020.00688 32850813 PMC7417319

[B8] Chen D. Wang M. Jiang X. Xiong Z. (2022). Comprehensive analysis of ZFPM2-AS1 prognostic value, immune microenvironment, drug sensitivity, and co-expression network: From gastric adenocarcinoma to pan-cancers. Discov. Oncol. 13, 24. 10.1007/s12672-022-00487-0 35416526 PMC9008104

[B9] Chen N. Zhu X. Zhu Y. Shi J. Zhang J. Tang C. (2021). The regulatory relationship and function of LncRNA fam225a-miR-206-ADAM12 in gastric cancer. Am. J. Transl. Res. 13 (8), 8632–8652. 34539984 PMC8430187

[B10] Chen T. Zhang C. Liu Y. Zhao Y. Lin D. Hu Y. (2019). A gastric cancer LncRNAs model for MSI and survival prediction based on support vector machine. BMC Genomics 20, 846. 10.1186/s12864-019-6135-x 31722674 PMC6854775

[B11] Dai L. Huang Z. Li W. (2021). Analysis of the PD-1 ligands among gastrointestinal cancer patients: Focus on cancer immunity. Front. Oncol. 11, 637015. 10.3389/fonc.2021.637015 33833994 PMC8021907

[B12] Dang S. Malik A. Chen J. Qu J. Yin K. Cui L. (2020). LncRNA SNHG15 contributes to immuno-escape of gastric cancer through targeting miR141/PD-L1. Onco Targets Ther. 13, 8547–8556. 10.2147/OTT.S251625 32943878 PMC7468375

[B13] Demaria O. Cornen S. Daëron M. Morel Y. Medzhitov R. Vivier E. (2019). Harnessing innate immunity in cancer therapy. Nature 574 (7776), 45–56. 10.1038/s41586-019-1593-5 31578484

[B14] Di Tomaso T. Mazzoleni S. Wang E. Sovena G. Clavenna D. Franzin A. (2010). Immunobiological characterization of cancer stem cells isolated from glioblastoma patients. Clin. Cancer Res. 16 (3), 800–813. 10.1158/1078-0432.CCR-09-2730 20103663 PMC2842003

[B15] Diao L. Wang S. Sun Z. (2018). Long noncoding RNA GAPLINC promotes gastric cancer cell proliferation by acting as a molecular sponge of miR-378 to modulate MAPK1 expression. Onco Targets Ther. 11, 2797–2804. 10.2147/OTT.S165147 29785127 PMC5957056

[B16] Erdogdu I. H. (2019). MHC class 1 and PDL-1 status of primary tumor and lymph node metastatic tumor tissue in gastric cancers. Gastroenterol. Res. Pract. 2019, 4785098. 10.1155/2019/4785098 30881447 PMC6381579

[B17] Galon J. Bruni D. (2019). Approaches to treat immune hot, altered and cold tumours with combination immunotherapies. Nat. Rev. Drug Discov. 18, 197–218. 10.1038/s41573-018-0007-y 30610226

[B18] Gaud G. Lesourne R. Love P. E. (2018). Regulatory mechanisms in T cell receptor signalling. Nat. Rev. Immunol. 18 (8), 485–497. 10.1038/s41577-018-0020-8 29789755

[B19] Goodman A. M. Kato S. Bazhenova L. Patel S. P. Frampton G. M. Miller V. (2017). Tumor mutational burden as an independent predictor of response to immunotherapy in diverse cancers. Mol. Cancer Ther. 16, 2598–2608. 10.1158/1535-7163.MCT-17-0386 28835386 PMC5670009

[B20] Gordon S. R. Maute R. L. Dulken B. W. Hutter G. George B. M. McCracken M. N. (2017). PD-1 expression by tumour-associated macrophages inhibits phagocytosis and tumour immunity. Nature 545, 495–499. 10.1038/nature22396 28514441 PMC5931375

[B21] Guo T. Wang W. Ji Y. Zhang M. Xu G. Lin S. (2021). LncRNA PROX1-AS1 facilitates gastric cancer progression via miR-877-5p/PD-L1 Axis. Cancer Manag. Res. 13, 2669–2680. 10.2147/CMAR.S275352 33776485 PMC7989960

[B22] He Y. Wang X. (2020). Identification of molecular features correlating with tumor immunity in gastric cancer by multi-omics data analysis. Ann. Transl. Med. 8, 1050. 10.21037/atm-20-922 33145269 PMC7575957

[B23] Jardim D. L. Goodman A. de Melo Gagliato D. Kurzrock R. (2021). The challenges of tumor mutational burden as an immunotherapy biomarker. Cancer Cell 39, 154–173. 10.1016/j.ccell.2020.10.001 33125859 PMC7878292

[B24] Jiang Q. Chen H. Tang Z. Sun J. Ruan Y. Liu F. (2021). Stemness-related LncRNA pair signature for predicting therapy response in gastric cancer. BMC Cancer 21, 1067. 10.1186/s12885-021-08798-1 34587919 PMC8482617

[B25] Joshi S. S. Badgwell B. D. (2021). Current treatment and recent progress in gastric cancer. CA Cancer J. Clin. 71, 264–279. 10.3322/caac.21657 33592120 PMC9927927

[B26] Kang Y. K. Boku N. Satoh T. Ryu M. H. Chao Y. Kato K. (2017). Nivolumab in patients with advanced gastric or gastro-oesophageal junction cancer refractory to, or intolerant of, at least two previous chemotherapy regimens (ONO-4538-12, ATTRACTION-2): A randomised, double-blind, placebo-controlled, phase 3 trial. Lancet 390, 2461–2471. 10.1016/S0140-6736(17)31827-5 28993052

[B27] Kulski J. K. (2019). Long noncoding RNA *HCP5*, a hybrid HLA class I endogenous retroviral gene: Structure, expression, and disease associations. Cells 8 (5), 480. 10.3390/cells8050480 31137555 PMC6562477

[B28] Lei L. Li N. Yuan P. Liu D. (2022). A new risk model based on a 11-m (6)A-related lncRNA signature for predicting prognosis and monitoring immunotherapy for gastric cancer. BMC Cancer 22, 365. 10.1186/s12885-021-09062-2 35382776 PMC8981748

[B29] Li J. Xiang R. Song W. Wu J. Kong C. Fu T. (2022). A novel ferroptosis-related LncRNA pair prognostic signature predicts immune landscapes and treatment responses for gastric cancer patients. Front. Genet. 13, 899419. 10.3389/fgene.2022.899419 35795206 PMC9250987

[B30] Liang X. Yu G. Zha L. Guo X. Cheng A. Qin C. (2021). Identification and comprehensive prognostic analysis of a novel chemokine-related lncRNA signature and immune landscape in gastric cancer. Front. Cell Dev. Biol. 9, 797341. 10.3389/fcell.2021.797341 35096827 PMC8795836

[B31] Liu R. Yang X. (2021). LncRNA LINC00342 promotes gastric cancer progression by targeting the miR-545-5p/CNPY2 Axis. BMC Cancer 21 (1), 1163. 10.1186/s12885-021-08829-x 34715819 PMC8556989

[B32] Luo Y. Yang J. Yu J. Liu X. Yu C. Hu J. (2020). Long non-coding RNAs: Emerging roles in the immunosuppressive tumor microenvironment. Front. Oncol. 10, 48. 10.3389/fonc.2020.00048 32083005 PMC7005925

[B33] Ma E. Hou S. Wang Y. Xu X. Wang Z. Zhao J. (2021). Identification and validation of an immune-related lncRNA signature to facilitate survival prediction in gastric cancer. Front. Oncol. 11, 666064. 10.3389/fonc.2021.666064 34760687 PMC8573392

[B35] Ma L. Zhou Y. Luo X. Gao H. Deng X. Jiang Y. (2017). Long non-coding RNA XIST promotes cell growth and invasion through regulating miR-497/MACC1 Axis in gastric cancer. Oncotarget 8 (3), 4125–4135. 10.18632/oncotarget.13670 27911852 PMC5354817

[B36] Marabelle A. Fakih M. Lopez J. Shah M. Shapira-Frommer R. Nakagawa K. (2020). Association of tumour mutational burden with outcomes in patients with advanced solid tumours treated with pembrolizumab: Prospective biomarker analysis of the multicohort, open-label, phase 2 KEYNOTE-158 study. Lancet Oncol. 21, 1353–1365. 10.1016/S1470-2045(20)30445-9 32919526

[B37] Miranda A. Hamilton P. T. Zhang A. W. Pattnaik S. Becht E. Mezheyeuski A. (2019). Cancer stemness, intratumoral heterogeneity, and immune response across cancers. Proc. Natl. Acad. Sci. U. S. A. 116, 9020–9029. 10.1073/pnas.1818210116 30996127 PMC6500180

[B38] Mu L. Wang Y. Su H. Lin Y. Sui W. Yu X. (2021). HIF1A-AS2 promotes the proliferation and metastasis of gastric cancer cells through miR-429/PD-L1 Axis. Dig. Dis. Sci. 66, 4314–4325. 10.1007/s10620-020-06819-w 33555514

[B39] Nai A. Zeng H. Wu Q. He Z. Zeng S. Bashir S. (2022). lncRNA/miR-29c-Mediated high expression of LOX can influence the immune status and chemosensitivity and can forecast the poor prognosis of gastric cancer. Front. Cell Dev. Biol. 9, 760470. 10.3389/fcell.2021.760470 35047494 PMC8762233

[B40] Newsom-Davis T. E. Wang D. Steinman L. Chen P. F. T. Wang L. X. Simon A. K. (2009). Enhanced immune recognition of cryptic glycan markers in human tumors. Cancer Res. 69 (5), 2018–2025. 10.1158/0008-5472.CAN-08-3589 19223535 PMC2657676

[B41] Okazaki T. Honjo T. (2007). PD-1 and PD-1 ligands: From discovery to clinical application. Int. Immunol. 19, 813–824. 10.1093/intimm/dxm057 17606980

[B42] Ou J. Lei P. Yang Z. Yang M. Luo L. Mo H. (2021). LINC00152 mediates CD8^+^ T-cell infiltration in gastric cancer through binding to EZH2 and regulating the CXCL9, 10/CXCR3 axis. J. Mol. Histol. 52 (3), 611–620. 10.1007/s10735-021-09967-z 33709190

[B43] Pardoll D. M. (2012). The blockade of immune checkpoints in cancer immunotherapy. Nat. Rev. Cancer 12, 252–264. 10.1038/nrc3239 22437870 PMC4856023

[B44] Qi P. Xu M. D. Shen X. H. Ni S. J. Huang D. Tan C. (2015). Reciprocal repression between TUSC7 and miR-23b in gastric cancer. Int. J. Cancer 137 (6), 1269–1278. 10.1002/ijc.29516 25765901

[B45] Reck M. Remon J. Hellmann M. D. (2022). First-Line immunotherapy for non-small-cell lung cancer. J. Clin. Oncol. 40, 586–597. 10.1200/JCO.21.01497 34985920

[B46] Reinke S. Bröckelmann P. J. Iaccarino I. Garcia-Marquez M. A. Borchmann S. Jochims F. (2020). Tumor and microenvironment response but no cytotoxic T-cell activation in classic Hodgkin lymphoma treated with anti-PD1. Blood 136, 2851–2863. 10.1182/blood.2020008553 33113552

[B47] Rizzo A. Mollica V. Ricci A. D. Maggio I. Massucci M. Rojas Limpe F. L. (2020). Third- and later-line treatment in advanced or metastatic gastric cancer: A systematic review and meta-analysis. Future Oncol. 16, 4409–4418. 10.2217/fon-2019-0429 31793342

[B48] Schreiber R. D. Old L. J. Smyth M. J. (2011). Cancer immunoediting: Integrating immunity's roles in cancer suppression and promotion. Science 331, 1565–1570. 10.1126/science.1203486 21436444

[B49] Simon A. K. Gallimore A. Jones E. Sawitzki B. Cerundolo V. Screaton G. R. (2002). Fas ligand breaks tolerance to self-antigens and induces tumor immunity mediated by antibodies. Cancer Cell 2 (4), 315–322. 10.1016/s1535-6108(02)00151-4 12398895

[B50] Smyth E. C. Nilsson M. Grabsch H. I. van Grieken N. C. Lordick F. (2020). Gastric cancer. Lancet 396, 635–648. 10.1016/S0140-6736(20)31288-5 32861308

[B51] Sun J. Jiang Q. Chen H. Zhang Q. Zhao J. Li H. (2021). Genomic instability-associated lncRNA signature predicts prognosis and distinct immune landscape in gastric cancer. Ann. Transl. Med. 9, 1326. 10.21037/atm-21-3569 34532463 PMC8422092

[B52] Sung H. Ferlay J. Siegel R. L. Laversanne M. Soerjomataram I. Jemal A. (2021). Global cancer statistics 2020: GLOBOCAN estimates of incidence and mortality worldwide for 36 cancers in 185 countries. CA Cancer J. Clin. 71, 209–249. 10.3322/caac.21660 33538338

[B53] Toor S. M. Murshed K. Al-Dhaheri M. Khawar M. Abu Nada M. Elkord E. (2019). Immune checkpoints in circulating and tumor-infiltrating CD4(+) T cell subsets in colorectal cancer patients. Front. Immunol. 10, 2936. 10.3389/fimmu.2019.02936 31921188 PMC6928042

[B54] Wang C-J. Chun-Chao Z. Jia X. Wang M. Zhao W. Y. Liu Q. (2019). The lncRNA UCA1 promotes proliferation, migration, immune escape and inhibits apoptosis in gastric cancer by sponging anti-tumor miRNAs. Mol. Cancer 18, 115. 10.1186/s12943-019-1032-0 31272462 PMC6609402

[B55] Wang F. H. Zhang X. T. Li Y. F. Tang L. Qu X. J. Ying J. E. (2021). The Chinese Society of Clinical Oncology (CSCO): Clinical guidelines for the diagnosis and treatment of gastric cancer, 2021. Cancer Commun. (Lond) 41, 747–795. 10.1002/cac2.12193 34197702 PMC8360643

[B56] Wang G. Duan P. Liu F. Wei Z. (2021). Long non-coding RNA CASC7 suppresses malignant behaviors of breast cancer by regulating miR-21-5p/FASLG axis. Bioengineered 12 (2), 11555–11566. 10.1080/21655979.2021.2010372 34889164 PMC8809951

[B57] Wang J. Wang B. Zhou B. Chen J. Qi J. Shi L. (2022). A novel immune-related lncRNA pair signature for prognostic prediction and immune response evaluation in gastric cancer: A bioinformatics and biological validation study. Cancer Cell Int. 22 (1), 69. 10.1186/s12935-022-02493-2 35144613 PMC8832759

[B58] Wang J. B. Li P. Liu X. L. Zheng Q. L. Ma Y. B. Zhao Y. J. (2020). An immune checkpoint score system for prognostic evaluation and adjuvant chemotherapy selection in gastric cancer. Nat. Commun. 11 (1), 6352. 10.1038/s41467-020-20260-7 33311518 PMC7732987

[B59] Wang J. Yu Z. Wang J. Shen Y. Qiu J. Zhuang Z. (2020). LncRNA NUTM2A-AS1 positively modulates TET1 and HIF-1A to enhance gastric cancer tumorigenesis and drug resistance by sponging miR-376a. Cancer Med. 9, 9499–9510. 10.1002/cam4.3544 33089970 PMC7774746

[B60] Wang W. Pei Q. Wang L. Mu T. Feng H. (2022). Construction of a prognostic signature of 10 autophagy-related lncRNAs in gastric cancer. Int. J. Gen. Med. 15, 3699–3710. 10.2147/IJGM.S348943 35411177 PMC8994655

[B61] Wang Y. Zhang X. Dai X. He D. (2021). Applying immune-related lncRNA pairs to construct a prognostic signature and predict the immune landscape of stomach adenocarcinoma. Expert Rev. Anticancer Ther. 21, 1161–1170. 10.1080/14737140.2021.1962297 34319826

[B62] Wang Y. Zhu G. Q. Tian D. Zhou C. W. Li N. Feng Y. (2022). Comprehensive analysis of tumor immune microenvironment and prognosis of m6A-related lncRNAs in gastric cancer. BMC Cancer 22, 316. 10.1186/s12885-022-09377-8 35331183 PMC8943990

[B63] Wang Y. Zhu G. Q. Tian D. Zhou C. W. Li N. Feng Y. (2022). Comprehensive analysis of tumor immune microenvironment and prognosis of m6A-related lncRNAs in gastric cancer. BMC Cancer 22 (1), 316. 10.1186/s12885-022-09377-8 35331183 PMC8943990

[B64] Wang Z. Cao L. Zhou S. Lyu J. Gao Y. Yang R. (2022). Construction and validation of a novel pyroptosis-related four-lncRNA prognostic signature related to gastric cancer and immune infiltration. Front. Immunol. 13, 854785. 10.3389/fimmu.2022.854785 35392086 PMC8980360

[B66] Wei G. H. Wang X. (2017). lncRNA MEG3 inhibit proliferation and metastasis of gastric cancer via P53 signaling pathway. Eur. Rev. Med. Pharmacol. Sci. 21 (17), 3850–3856. 28975980

[B67] Wei L. Sun J. Zhang N. Zheng Y. Wang X. Lv L. (2020). Noncoding RNAs in gastric cancer: Implications for drug resistance. Mol. Cancer 19, 62. 10.1186/s12943-020-01185-7 32192494 PMC7081551

[B68] Wei Z. Chen L. Meng L. Han W. Huang L. Xu A. (2020). LncRNA HOTAIR promotes the growth and metastasis of gastric cancer by sponging miR-1277-5p and upregulating COL5A1. Gastric Cancer 23 (6), 1018–1032. 10.1007/s10120-020-01091-3 32583079

[B69] Wen-Ming Chen Huang Ming-de Kong Rong Xu T. P. Zhang E. B. Xia R. (2015). Antisense long noncoding RNA HIF1A-AS2 is upregulated in gastric cancer and associated with poor prognosis. J. .Dig Dis. Sci. 60, 1655–1662. 10.1007/s10620-015-3524-0 25686741

[B70] Winograd P. Hou S. Court C. M. Lee Y. T. Chen P. J. Zhu Y. (2020). Hepatocellular carcinoma-circulating tumor cells expressing PD-L1 are prognostic and potentially associated WithResponse to checkpoint inhibitors. Hepatol. Commun. 4, 1527–1540. 10.1002/hep4.1577 33024921 PMC7527695

[B71] Xiao S. Liu X. Yuan L. Wang F. (2021). A ferroptosis-related lncRNAs signature predicts prognosis and therapeutic response of gastric cancer. Front. Cell Dev. Biol. 9, 736682. 10.3389/fcell.2021.736682 34926441 PMC8674955

[B73] Xiao X. Cheng W. Zhang G. Wang C. Sun B. Zha C. (2022). Long noncoding RNA: Shining stars in the immune microenvironment of gastric cancer. Front. Oncol. 12, 862337. 10.3389/fonc.2022.862337 35402261 PMC8989925

[B75] Xie C. Guo Y. Lou S. (2020). LncRNA ANCR promotes invasion and migration of gastric cancer by regulating FoxO1 expression to inhibit macrophage M1 polarization. Dig. Dis. Sci. 65 (10), 2863–2872. 10.1007/s10620-019-06019-1 31894487

[B76] Xie J. Fu L. Jin L. (2021). Immunotherapy of gastric cancer: Past, future perspective and challenges. Pathol. Res. Pract. 218, 153322. 10.1016/j.prp.2020.153322 33422778

[B77] Xiong G. Yang L. Chen Y. Fan Z. (2015). Linc-POU3F3 promotes cell proliferation in gastric cancer via increasing T-reg distribution. Am. J. Transl. Res. 7 (11), 2262–2269. 26807174 PMC4697706

[B78] Yu J. Fang C. Zhang Z. Zhang G. Shi L. Qian J. (2020). H19 rises in gastric cancer and exerts a tumor-promoting function via miR-138/E2F2 Axis. Cancer Manag. Res. 12, 13033–13042. 10.2147/CMAR.S267357 33376397 PMC7762430

[B79] Yu P. Wang Y. Yu Y. Wang A. Huang L. Zhang Y. (2021). Deep targeted sequencing and its potential implication for cancer therapy in Chinese patients with gastric adenocarcinoma. Oncologist 26, e756–e768. 10.1002/onco.13695 33511732 PMC8100567

[B80] Yuan L. Xu Z. Y. Ruan S. M. Mo S. Qin J. J. Cheng X. D. (2020). Long non-coding RNAs towards precision medicine in gastric cancer: Early diagnosis, treatment, and drug resistance. Mol. Cancer 19, 96. 10.1186/s12943-020-01219-0 32460771 PMC7251695

[B81] Zeng C. Liu Y. He R. Lu X. Dai Y. Qi G. (2022). Identification and validation of a novel cellular senescence-related lncRNA prognostic signature for predicting immunotherapy response in stomach adenocarcinoma. Front. Genet. 13, 935056. 10.3389/fgene.2022.935056 36092903 PMC9453157

[B82] Zhang L. Wang Y. Li Z. Lin D. Liu Y. Zhou L. (2021). Clinicopathological features of tumor mutation burden, Epstein-Barr virus infection, microsatellite instability and PD-L1 status in Chinese patients with gastric cancer. Diagn Pathol. 16, 38. 10.1186/s13000-021-01099-y 33933102 PMC8088709

[B83] Zhou J. Zhi X. Wang L. Wang W. Li Z. Tang J. (2015). Linc00152 promotes proliferation in gastric cancer through the EGFR-dependent pathway. J. Exp. Clin. Cancer Res. 34, 135. 10.1186/s13046-015-0250-6 26538117 PMC4632266

[B84] Zhu K. Ren Q. Zhao Y. (2019). lncRNA MALAT1 overexpression promotes proliferation, migration and invasion of gastric cancer by activating the PI3K/AKT pathway. Oncol. Lett. 17 (6), 5335–5342. 10.3892/ol.2019.10253 31186750 PMC6507354

[B85] Zou Y. Chen B. (2021). Long non-coding RNA HCP5 in cancer. Clin. Chim. Acta 512, 33–39. 10.1016/j.cca.2020.11.015 33245911

