# Supplementary material for: LncRNA and its role in gastric cancer immunotherapy
Source: Front Cell Dev Biol. 2023 Feb 16;11:1052942. doi: 10.3389/fcell.2023.1052942 (PMC9978521; doi:10.3389/fcell.2023.1052942)
Supplement: Supplementary file 1 [file Table1.DOCX]

Table 1 LncRNAs involved in TIME of GC disease

| LncRNA | Pathway | Type of disease | Reference |
| --- | --- | --- | --- |
| LINC00001 | miR-497/MACC1 axis | GC | [77] |
| LINC00008 | miR-138/E2F2 axis | GC | [78] |
| LINC00023 | p53 signaling pathway | GC | [79] |
| LINC00047 | PI3K/AKT pathway | GC | [80] |
| LINC00152 | EGFR-dependent pathway | GC | [81] |
| LINC00256A | FAM225A-miR-206-ADAM12 axis | GC | [82] |
| LINC00342 | miR-545-5p/CNPY2 axis | GC | [83] |
| LINC00902 | p53 and mIR-23b | GC | [84] |
| LINC01540 | miR-378 to modulate MAPK1 expression | GC | [85] |
| LINC -POU3F3 | TGF-beta signal pathway | GC | [86] |
